# Quercetin Inhibits Inflammasome Activation by Interfering with ASC Oligomerization and Prevents Interleukin-1 Mediated Mouse Vasculitis

**DOI:** 10.1038/srep41539

**Published:** 2017-02-02

**Authors:** Talita P. Domiciano, Daiko Wakita, Heather D. Jones, Timothy R. Crother, Waldiceu A. Verri, Moshe Arditi, Kenichi Shimada

**Affiliations:** 1 Department of Pathology, Center of Biological Science, Londrina State University, Londrina, Paraná, Brazil; 2 Department of Pediatric, Infectious diseases and Immunology, Cedars-Sinai Medical Center, Los Angeles, California, USA; 3 Division of Pulmonary and Critical Care Medicine, Cedars-Sinai Medical Center, Los Angeles, California, USA

## Abstract

Interleukin-1β (IL-1β) is a highly inflammatory cytokine that significantly contributes to both acute and chronic inflammatory diseases. The secretion of IL-1β requires a unique protease, caspase-1, which is activated by various protein platforms called inflammasomes. Data suggests a key role for mitochondrial reactive oxygen species for inflammasome activation. Flavonoids constitute a group of naturally occurring polyphenolic molecules with many biological activities, including antioxidant effects. In this study, we investigated the effect of three flavonoids, quercetin (QUC), naringenin, and silymarim on inflammasome activation. We found that QUC inhibits IL-1β secretion by both the NLRP3 and AIM2 inflammasome in a dose dependent manner, but not the NLRC4 inflammasome. QUC inhibition of the inflammasome was still observed in *Atg16l1* knockout macrophages, indicating that QUC’s effect was autophagy independent. Since QUC inhibited both NLRP3 and AIM2 inflammasomes but not NLRC4, we assessed ASC speck formation. QUC reduced ASC speck formation and ASC oligomerization compared with controls. Additionally, QUC inhibited IL-1β in Cryopyrin-Associated Periodic Syndromes (CAPS) macrophages, where NLRP3 inflammasome is constitutively activated. In conclusion, QUC inhibits both the NLRP3 and AIM2 inflammasome by preventing ASC oligomerization and may be a potential therapeutic candidate for Kawasaki disease vasculitis and other IL-1 mediated inflammatory diseases.

Inflammation is a fundamental multi-step cellular response to harmful stimuli such as pathogens, toxins, trauma, or heat injury. Thus, it can be considered that a major role of the immune system is to maintain homeostatic tissue function. However, if inflammation goes on unchecked, sustained immune responses can lead to serious host inflammatory injury and various diseases. Increased IL-1β, locally or systemic, has been linked to a number of human hereditary or acquired diseases, and antagonists of IL-1β or its receptor are increasingly being used successfully for treatments for a number of these inflammatory diseases such as cryopyrin-associated periodic syndromes (CAPS), gout, atherosclerosis, type II diabetes and even in Kawasaki disease vasculitis (KD)^1^,^2^.

The inflammasomes are multimeric protein complexes that consist of a sensor molecule, the adaptor protein ASC and capspase-1 via Caspase activation and recruitment domains (CARD)-CARD interactions^3^, which are induced by the activation of pattern recognition receptors (PPRs) resulting in the release of highly pro-inflammatory cytokines IL-1β and IL-18^4^. Apoptosis-associated speck like protein containing a CARD (ASC), encoded by *Pycard*, is a cytosolic protein and can control the activation of caspase-1, bridging NLR family, pyrin domain containing 3 (NLRP3) and Absent in Melanoma 2 (AIM2) inflammasomes by self-assembly into fiber-like structures such as ASC specks^4^. Whether ASC is also required to NLR family, CARD domain containing 4 (NLRC4) inflammasome activation is less clear^5^.

Kawasaki disease is a multisystem acute vasculitis that primarily affects young children and is the most common acquired cardiovascular disease among children in developed countries^6^. Without treatment, 25% of KD patient develop heart disease involving coronary aneurysms and dilatations^1^,^7^,^8^. While sequelae of arterial inflammation in the acute phase of KD are generally self-limiting and well documented, its late effects, such as cardiovascular complication, can be life-threatening^9^. A mouse model of Kawasaki Diseases vasculitis and coronary arteritis is available that closely mimics the important histological features of the coronary artery lesions seen in patients with KD^10^. Lehman *et al*. reported in 1985 that a single i.p. injection of a cell wall extract from *Lactobacillus casei* (LCWE), reproducibly induces aortitis and proximal coronary arteritis that are histopathologically very similar to the coronary arteritis (CA) observed in human KD. Our group has recently shown that NLRP3 inflammasome activation and IL-1β are critically important in the development of coronary arteritis and abdominal aorta aneurysms (AAA) and dilatations seen in an experimental Kawasaki disease vasculitis mouse model^1^,^11^.

Quercetin is a dietary flavonol widely found in fruits, vegetables, and nuts. Among polyphenols quercetin is one of the most potent anti-oxidants as demonstrated in different studies. Quercetin inhibits oxidative species generating enzymes such as xanthine oxidase, LOX, and nicotinamide adenine dinucleotide phosphate oxidase (NADPH)^12^,^13^,^14^. It is a potent anti-cancer agent, exhibiting different activities such as cell cycle regulation, interaction with type II estrogen binding sites, and tyrosine kinase inhibition^15^. Silymarin, a flavonol complex extracted from the seeds of the milk thistle plant (*Silybum marianum*), an effective antioxidant, increases glutathione (GSH) in liver cells and provides important protective activities against oxidative stress^16^. Silymarin has proven to possess potent anti-inflammatory activity by inhibition of MAPKs and NF-κB pathways^17^. Naringenin, another flavonoid belonging to the flavanones group, has various impressive pharmacological activities including antioxidant, antimicrobial, anti-inflammatory, and anticancer activity^18^.

The molecular mechanism underlying the anti-inflammatory activity of quercetin, silymarin and naringenin in the context of KD is not completely understood. Therefore, in this study we focused on the immuno-pharmacologic mechanism of these flavonoids on inflammasome activated macrophages *in vitro*, as well as and a bacterial cell wall extract induced coronary arteritis and abdominal aorta aneurysm. Here we report that quercetin inhibited inflammasome activity through inhibition of ASC oligomerization *in vitro* and quercetin treatment was also beneficial in preventing vascular inflammation in the KD vasculitis mouse model, which is an IL-1-dependent experimental model.

## Results

### Quercetin inhibits NLRP3 and AIM2 inflammasome activation

**Figure 1:**
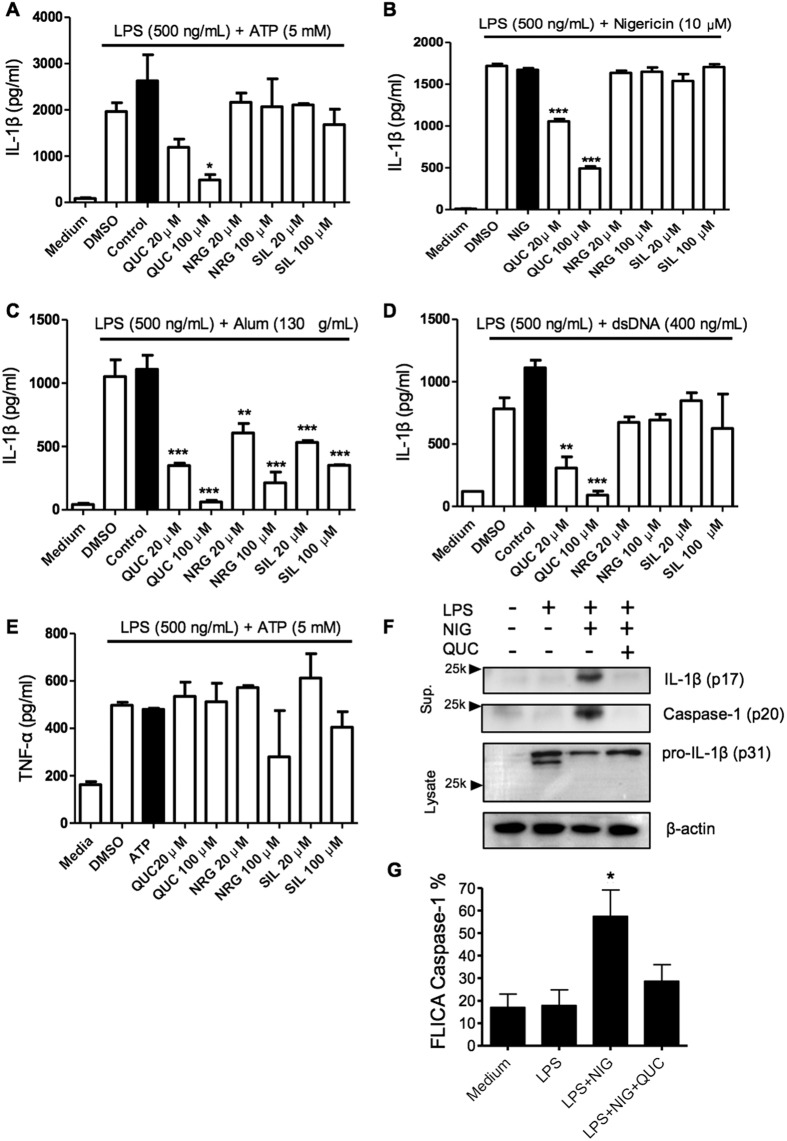
Quercetin inhibits NLRP3 and AIM2 Inflammasomes. LPS (500 ng/ml)-primed BMDM were treated with the indicated flavonoids and concentrations or vehicle (DMSO 0.01%), then stimulated with (**A**) 5 mM ATP (**B**) 10 μM Nigericin (**C**) 130 μg/ml Alum or (**D**) 400 ng/ml Poly (dA:dT) 30 minutes after treatment. (**A**–**D**) IL-1β and (**E**) TNF-α concentration in the culture supernatant were measured by ELISA. (**B**,**F**,**G**) LPS-primed BMDM were stimulated with Nigericin treated with quercetin 30 minutes before secondary stimulation. Supernatants and Lysate of BMDM were analyzed by immunoblotting. Cells were stained with FLICA and Caspase-1 activation was determined by FLICA positivity. Data shown are representative of two or more independent experiments (means ± SD) *p < 0.05, **p < 0.01, ***p < 0.001.

Inflammasome activation depends on 2 signals. The first, via NF-κB activation that leads to pro-IL-1β and pro-caspase-1 synthesis. The second signal is required for assembling the inflammasome complex, which recruits pro-caspase-1. The oligomerization of pro-caspase-1 triggers self-proteolysis to active caspase-1, which cleaves and releases mature IL-1β from the cell. While it is already known that flavonoids can potentially inhibit NF-κB activation^19^ thereby preventing pro-IL-1β and pro-caspase-1 synthesis, it is not known whether flavonoids could inhibit inflammasome activation by interfering with signal 2. To investigate this, BMDM were primed with LPS followed by stimulation with ATP, nigericin, or alum (NLRP3 activators), or stimulated with dsDNA for AIM2 inflammasome activation. To distinguish from signal 2 from signal 1, we first primed the BMDM with LPS, incubated for 3 h to allow pro-IL-1β production, and then followed with flavonoid treatment before secondary stimulation. We found that treatment with quercetin inhibited IL-1β secretion by NLRP3 and AIM2 inflammasomes in a dose dependent manner (Fig. 1A–D). Interestingly, naringenin and silymarin only inhibited the Alum induced NLRP3 inflammasome (Fig. 1A–D), suggesting that these flavonoids might be associated with lysosomal destabilization^20^. Importantly, under these conditions, quercetin treatment did not affect TNF-α production (Fig. 1E), suggesting that quercetin can inhibit IL-1β secretion by interfering with signal-2. Corroborating our secretion data, we observed more pro-IL-1β in the quercetin treated cell lysate compared with control and a reduced amount mature IL-1β in the quercetin treated cells. In addition, quercetin reduced Caspase-1 activity observed in LPS plus nigericin (Fig. 1F,G). These data also indicate that quercetin interfered with activation inflammasome and not IL-1β synthesis (Fig. 1F).

### Quercetin does not inhibit NLRC4 inflammasome activation

**Figure 2:**
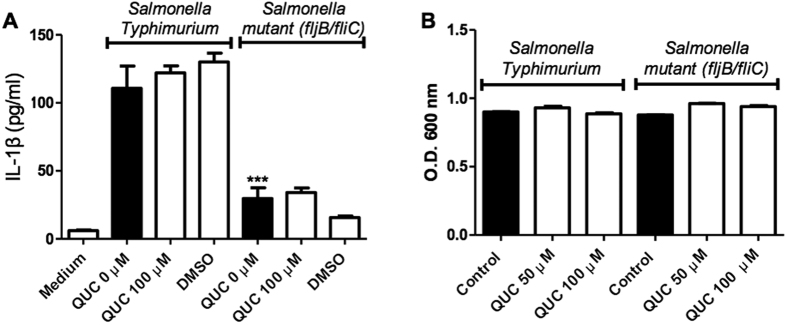
Quercetin does not inhibit NLRC4 inflammasome activation. (**A**) IL-1β concentrations (ELISA) in *Nlrp3*^−/−^ BMDM culture supernatants primed with LPS (500 ng/ml) for 2 h and stimulated with WT *Salmonella* or non-flagellated *Salmonella* Mutant (Δ*fljB/fliC*) (MOI 5) for 90 minutes and then treated with quercetin or vehicle (DMSO 0.01%) followed by 5 h incubation. (**B**) *Salmonella* growth in the presence of quercetin. O.D were measured after 8 h incubation. Data shown are representative of two or more experiments (means ± SD) ***p < 0.001.

Since quercetin was able to inhibit both the NLRP3 and AIM2 inflammasomes, we investigated if this flavonoid could also inhibit the NLRC4 inflammasome. Flagellate intracellular bacteria, such as *Salmonella typhimurium*, activate this pathway. NLRP3 and AIM2 contain a Pyrin domain (PYD) which interacts with the PYD of ASC. The CARD domain of ASC then binds the CARD of caspase-1 via CARD-CARD interaction serving as a bridge between pro-caspase-1 and pyrin-containing inflammasomes^21^. However, unlike NLRP3 and AIM2, NLRC4 contains its own CARD domain, which can interact directly with capase-1, thus making ASC dispensable for NLRC4-dependent caspase-1 activation^2^. Furthermore, ASC phosphorylation is not required in NLRC4 inflammasome while NLRP3 and AIM2 inflammasomes require ASC phosphorylation^22^. Despite the evidence the possibility remains that ASC may still be required for full NLRC4 activation in different conditions^23^. *S. typhinurium* induces both NLRP3 as well as NLRC4 inflammasomes^5^,^23^. To address if quercetin also inhibits NLRC4 inflammasome, we used *Nlrp3*^−/−^ BMDM. Additionally, we used type *S. typhinurium (St*) and mutant *St (St* ΔfljB/ΔfliC; non-flagellated) that does not induce NLRC4 inflammasome activation^5^. We found that wild type *St* induced IL-1β secretion in *Nlrp3*^−/−^ BMDM but was un-altered by quercetin. However, the *St* flagelin mutant significantly inhibited the IL-1β secretion irrespective of quercetin presence or absence (*P* < 0.001). These results indicates that quercetin does not inhibit NLRC4 inflammasome activation (Fig. 2A). To assure whether the lack of inhibition was not due to a possible antibiotic action we incubated both *St* strains with quercetin and observed its growth after 8 hours of incubation. The growth levels did not change with quercetin confirming there was no antibiotic effect (Fig. 2B).

### NLRP3 inflammasome inhibition by quercetin is independent of autophagy

**Figure 3:**
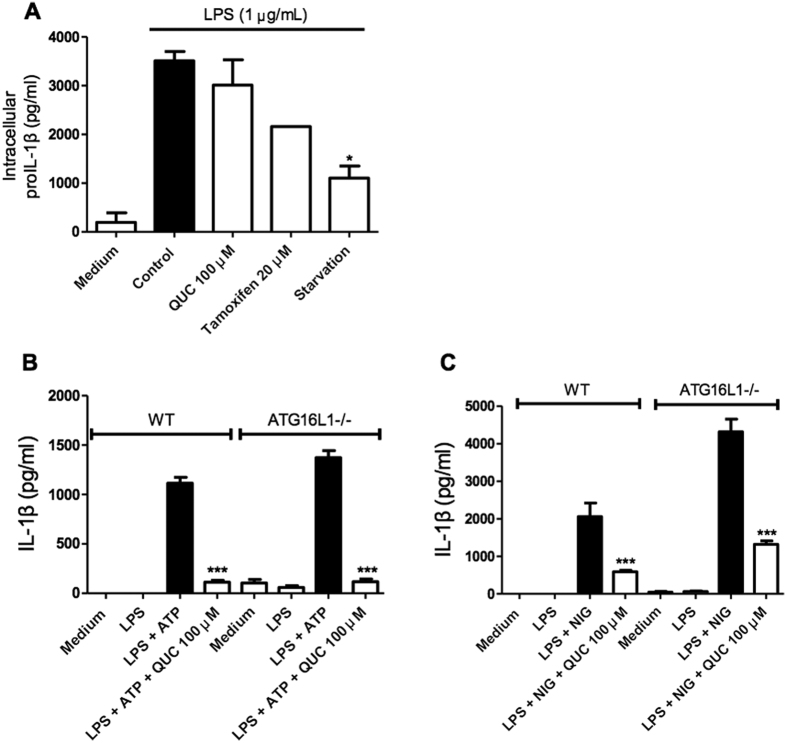
Autophagy is dispensable for NLRP3 inflammasome inhibition by Quercetin. (**A**) Intracellular pro-IL-1β concentrations (ELISA) in BMDM lysates. BMDM primed with LPS (500 ng/ml) were treated with quercetin, tamoxifen or serum-free medium. (**B**,**C**) Wild type and Atg16l1^fl/fl^ LysM^Cre^ BMDM primed with LPS (500 ng/ml) were stimulated with (**B**) ATP (5 mM) or (**C**) Nigericin (10 μM) and treated with quercetin 30 minutes before secondary stimulation. Data shown are representative of two or more independent experiments (means ± SD) *p < 0.05, ***p < 0.001.

It has been reported that quercetin can induce autophagy in gastric cancer cells and HeLa cells^24^,^25^. Autophagy is the process by which cellular components can be recycled, either as a normal process or to remove damaged organelles^26^. Autophagy can also compensate for cellular stress and inhibit NLRP3 inflammasome activation^27^,^28^. We next assessed whether quercetin affects pro-IL1β stability in BMDM in comparison to autophagy inducers. LPS-induced intracellular pro-IL-1β amounts did not change during quercetin treatment while both autophagy inducers, tamoxifen treatment and starvation, reduced intracellular pro-IL-1β levels (Fig. 3A), indicating that quercetin does not promote pro-IL-1β degradation (Fig. 3A). We also used *Atg16l1*^−/−^ BMDM to observe inflammasome activation. LPS-primed BMDM secreted IL-1β in response to ATP or nigericin stimulation in both wild type and autophagy deficient BMDM. However, quercetin still inhibited IL-1β secretion in both WT and *Atg16l1*^−/−^ BMDM (Fig. 3B,C) suggesting that inflammasome inhibition by quercetin does not involve autophagy.

### Quercetin inhibits ASC-speck formation

**Figure 4:**
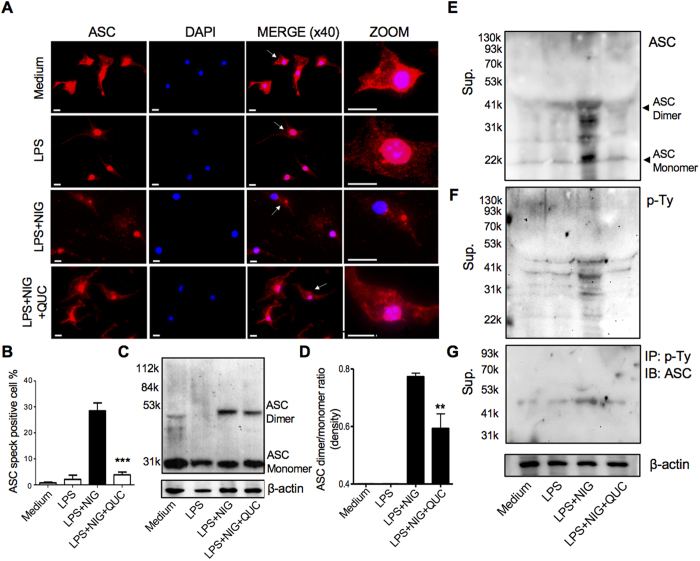
Quercetin inhibits ASC speck formation and oligomerization. LPS-primed BMDM were treated with quercetin 30 minutes before stimulation with nigericin (10 μM) and analyzed by Immunostaining (**A**). The white arrow represents ASC specks. Scale bar represents 10 μm. (**B**) Percentage of cells containing ASC speck. (**C**,**D**) Cross-linked lysate of BMDM were analyzed with anti-ASC immunoblotting (**C**) and quantified by densitmetry (**D**). Supernatants were analyzed by anti-ASC (**E**) and anti-phosphotyrosine immunoblotting (**F**). Supernatants were immunoprecipitated with anti-phosphotyrosine beads, and analyzed anti-ASC immunoblotting (**G**). Data shown are representative of two or more experiments (means ± SD) ***p < 0.001, **p < 0.01.

Since quercetin inhibited both the NLRP3 and AIM2 inflammasome, but not the NLRC4 inflammasome, we hypothesized that quercetin inhibition might be through the adaptor protein ASC. To investigate this possibility, we next visualized NLRP3 inflammasome complexes as ASC specks by immunofluorescence staining^29^. As previously reported^22^, after stimulation with nigericin, we also observed that the number of cells containing ASC specks increased (Fig. 4A,B). However, quercetin treated cells showed a significantly reduced number of ASC specks (Fig. 4A,B). In order to confirm this inhibition of ASC speck formation by quercetin, we assessed for ASC oligomerization (dimer and monomer forms) in the lysate by immunoblot. ASC dimers were detected in LPS + Nigericin stimulation, but the quercetin-treated cells had less ASC dimerization compared with controls (Fig. 4C,D). It has been reported that ASC is phosphorylated and then secreted in the supernatant^22^,^30^,^31^. We investigated whether the inhibition of inflammasome activation by quercetin is associated with ASC phosphorylation. We observed that secreted oligomerized ASC was phosphorylated and quercetin inhibited phosphorylated-ASC secretion (Fig. 4E–G). Interestingly, we observed ASC dimer in the supernatant without cross-linking although mostly ASC oligomerization were seen in cell lysate with prior protein cross-linker treatment (e.g. DSS). These data supported the irreversible biochemical changes of ASC occurs during inflammasome activation. Taken together, our data suggest that quercetin inhibits inflammasome activation by inhibiting ASC oligomerization.

### Quercetin inhibits constitutively active NLRP3 inflammasome

**Figure 5:**
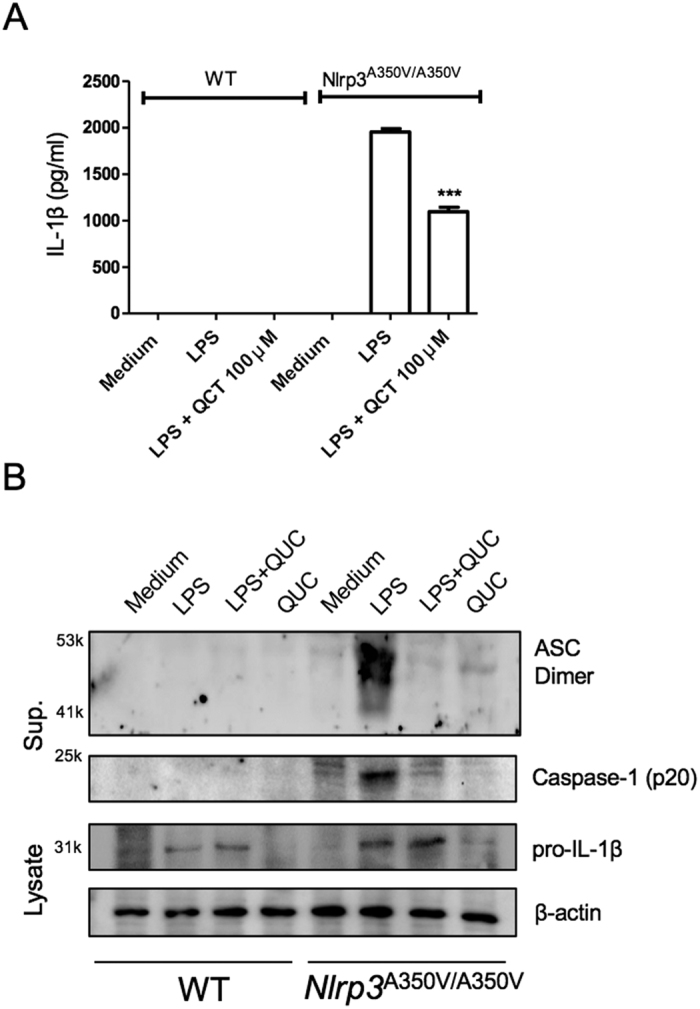
Quercetin inhibits auto-reactive NLRP3 inflammasome. (**A**) IL-1β concentrations in *Nlrp3*^*A350V/A350V*^ and WT BMDM culture supernatants primed with LPS (500 ng/ml) for 90 min and treated with quercetin 1 h after LPS. (**B**) Supernatants of BMDM were analyzed by anti-ASC, anti-Caspase-1 immunoblotting. Lysates of BMDM were analyzed by anti-IL-1β and anti-β-actin immunoblotting. Data shown are representative of two or more experiments (means ± SD) ***p < 0.001.

Cryopyrin-associated periodic syndrome (CAPS) is auto inflammatory disorder and associated with *Nlrp3* mutations^32^. Brydges *et al*.^33^ found that a specific mutation in BMDC resulted in NLRP3 activation and IL-1β secretion in response to only LPS; not requiring secondary stimulation such as ATP. Thus, these gain of function mutations are considered to result in a constitutively active inflammasome. Using NLRP3 mutant mice expressing Muckle-Wells syndrome (MWS) mutation at A350V we addressed if quercetin still could inhibit IL-1β secretion. As expected, WT BMDM stimulated with LPS were unable to secrete IL-1β (Fig. 5A). However, NLRP3^A350V^ BMDM did secrete IL-1β in response to LPS alone (Fig. 5A). Quercetin was also able to inhibit IL-1β secretion by NLRP3^A350V^ BMDM treated with LPS (Fig. 5A). Similar to the quercetin inhibition of ASC oligomerization induced by LPS+NIG in WT BMDM (Fig. 4), quercetin inhibited ASC oligomerization induced by LPS alone in NLRP3^A350V^ BMDM (Fig. 5B). These data suggest that quercetin inhibits inflammasome assembly events by inhibiting ASC oligomerization but not activation of NLRP3.

### Quercetin prevents mice from LCWE-Induced coronary arteritis and aortic aneurysms in experimental Kawasaki disease vasculitis model

**Figure 6:**
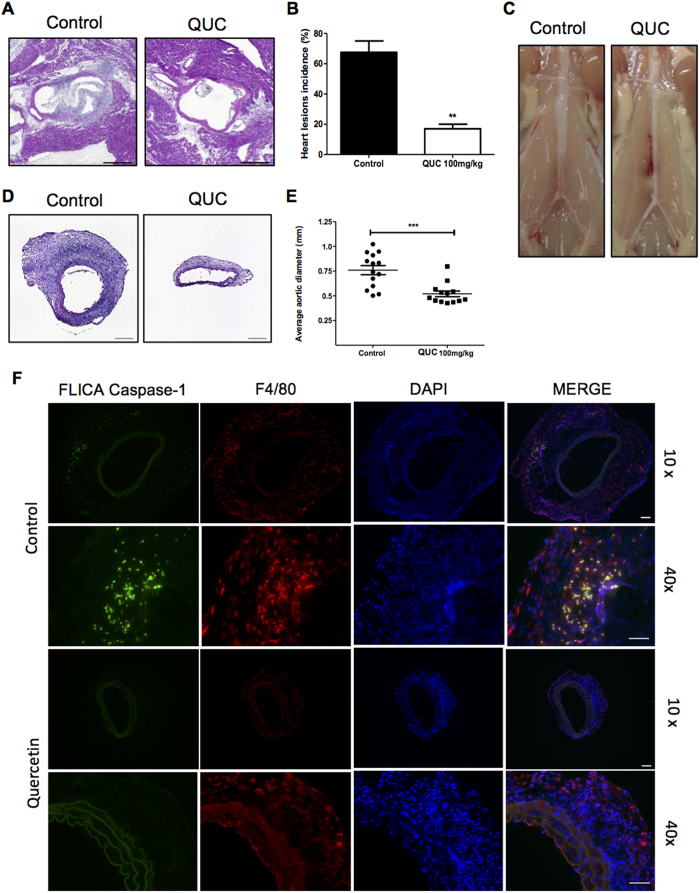
Quercetin prevents mice from LCWE-Induced coronary and aortic lesions. Wild type mice were administered 250 μg of LCWE i.p., treated daily with quercetin 100 mg/kg or vehicle (control), i.p. and their hearts and aorta were harvested on day 7. (**A**) Representative hematoxylin and eosin–stained heart sections (10 × - Scale bar 500 μm). (**B**) Heart lesion incidence. (**C**) Representative abdominal aorta. (**D**) Representative hematoxylin and eosin–stained aorta sections (10× – Scale bar 200 μm). (**E**) Average aortic diameter. (**F**) Representative aorta section immunostaining (10× – Scale bar 100 μm; 40X - Scale bar 50 μm). Data shown are representative of two or more experiments (mean ± S.E.M.) and were compared by use of the Tukey test (**B**,**E**). **p < 0.005 ***p < 0.001.

Since we demonstrated that the cardiovascular lesion development in the KD vasculitis mouse model is NLRP3 and IL-1-dependent^1^,^11^, we investigated whether quercetin could be beneficial for this KD vasculitis mouse model *in vivo*. We administered Quercetin i.p. (daily) in LCWE-injected mice. 7 days following LCWE injection, the hearts were collected and analyzed for coronary artery inflammation as described previously^1^. Quercetin treatment significantly reduced the incidence of coronary arteritis compared with controls (72% reduction) (Fig. 6A,B). In addition to blocking coronary arteritis, quercetin treatment inhibited AAA formation as measured by maximal abdominal aorta diameter and inflammatory histology (Fig. 6C–E). Quercetin treatment prevented the significant intimal proliferation and massive myofibroblastic proliferation observed in the LCWE-induced vasculitis mice (27% reduction) (Fig. 6D). We next determined if the inflammasome was activated locally in the vascular tissues in the LCWE-induced vasculitis mice. For this purpose, we visualized Casapse-1 activity in the vascular lesions using the fluorescent inhibitor of caspases (FLICA) assay. Active caspase-1 has previously been identified in macrophages in the coronary and AAA lesions observed in this LCWE-induced vasculitis model (unpublished data)^11^. As expected, FLICA-positive macrophages were observed in the abdominal aorta aneurysm lesions of the LCWE-injected mice (Fig. 6F). Importantly, quercetin completely inhibited this local caspase-1 activity in the LCWE-induced vascular lesions (Fig. 6F). Altogether, these results suggest that quercetin treatment may be beneficial to prevent vascular inflammation and vasculitis in this KD mouse model or other inflammasome/IL-1-mediated inflammatory diseases.

## Discussion

The inflammasome is a multiprotein oligomer consisting of caspase-1, ASC, and NLRs that regulates maturation of IL-1β and IL-18^3^. Inflammasome activation is required for many inflammatory processes and its activation generally requires two separate signals. NF-κB activation (signal 1), resulting from signaling such as TLRs, induces pro-IL-1β and pro-IL-18 production. The second signal is often a danger signal or a form of cellular stress. To date, the mitochondria and mitochondrial ROS has emerged as a central hub for NLRP3 inflammasome activation^34^. Additionally, lysosomal damage and cytosolic K^+^ efflux have been implicated in NLRP3 activation^35^. In addition to their ability to inhibit NF-κB activation, flavonoids can scavenge ROS, thus we evaluated quercetin, naringenin, and silymarin in their ability to inhibit the second signal and inflammasome activation. We found that both ATP and nigericin (inducers of mitochondrial dysfunction and K^+^ efflux), as well as alum (lysosomal damage) induced NLRP3 inflammasome activation were inhibited by quercetin. Similarly, quercetin also inhibited cholesterol crystal induced NLRP3 inflammasome activation. A previous study suggests that due to similar structures with allopurinol, quercetin and rutin were efficacious in reversing fructose-induced renal NLRP3 inflammasome activation in rats^36^. Their proposed mechanism was by inhibiting signal 1. However, our data suggests that in addition to inhibiting signal 1, quercetin can also directly inhibit the inflammasome component ASC assembly in macrophages as evidenced by the inhibition by of a constitutively active NLRP3 inflammasome. We also observed that quercetin inhibited dsDNA-induced AIM2 inflammasome, but not flagellin-induced NLRC4 inflammasome. Our data suggests that the signal 2 inhibition by quercetin may act only on ASC dependent inflammasomes.

During inflammasome activation, autophagy is induced in parallel^37^. Activation of autophagy itself inhibits the activation of NLRP3 inflammasome, likely through the prevention of apoptosis^38^ and or mitochondrial dysfunction^34^. Class III PI3K induces autophagy in a complex with Beclin 1, and thus class III PI3K inhibitors, such as wortmannin inhibits autophagy^39^. Walker *et al*. demonstrated that quercetin and wortmannin, a steroid metabolite of the fungi *Penicillium funiculosum*, possess important structure similarities and thus acts as inhibitors of phosphoinositide 3-kinases (PI3Ks)^40^. Although, quercetin may potentially be able to block autophagy, we observed that quercetin inhibition of IL-1β secretion occurred normally in ATG16L1 deficient BMDM, suggesting the inflammasome inhibition by quercetin occurs in an autophagy independent manner.

Upon activation of NLRP3, ASC proteins assemble into large fiber-like structures that amplifies caspase-1 activation. Baroja-Mazo *et al*.^41^ recently showed ASC-speck oligomerization 30 minutes after NLPR3 inflammasome activation. They also found that both ASC and the structural, gain-of-function, CAPS-associated NLRP3 mutant pD303N oligomerized into active particles detected in the serum of patients with CAPS. Previous studies show that NLRP3 mutant patients correlated with the aggregation of NLRP3 into particles with pro-inflammatory extracellular activity that induced the release of ASC specks^42^. In addition, another study found that during active disease, patients with CAPS have enhanced serum concentrations of ASC oligomers^41^. Indeed, in the current study, we discovered a novel mechanism by which quercetin blocks IL-1 production, through the inhibition of ASC-speck formation in BMDM, directly blocking the activation of the NLRP3 inflammasome. Thus, in addition to inhibiting signal 1, quercetin directly inhibits inflammasome activation by preventing ASC oligomerization. Additional studies are required to further understand the exact molecular mechanisms of how quercetin blocks ASC oligomerization. According to previous report by Hara *et al*., JNK (*Mapk8*) and Syk are important to phosphorylation on ASC Tyr144 which is required for NLRP3 and AIM2 inflammasome activation^22^. One possible mechanism may be that quercetin inhibits Syk and/or JNK, and inhibits phosphorylation of ASC^43^,^44^. However, it remains unclear how it works *in vitro* as quercetin was added after LPS stimulation (signal 1). Interestingly, naringenin and silymarin may not be able to protect JNK or protect partially in macrophages^45^,^46^, which may explain why naringenin and silymarin did not inhibit ATP and NIG-induced NLRP3 inflammasome and AIM2 inflammasome but quercetin did (Fig. 1).

Quercetin is already known to exert immune and inflammation modulating activity in several biological and experimental murine models and as well as an inverse association between quercetin intake and coronary heart disease^47^. Lara-Guzman *et al*. demonstrated that dyslipidemic *Apoe*^−/−^ mice treated with quercetin had significant reduction in atherosclerosis^48^. The ability of quercetin to inhibit signal 1 and prevent ASC oligomerization, directly inhibiting NLRP3 inflammasome activation make this molecule a potential therapeutic agent in inflammasome-mediated disorders. Using a mouse model of LCWE-induced vasculitis, which is dependent on IL-1β and inflammasome activation, we observed that quercetin significantly inhibited the cardiovascular lesions in the LCWE-induced vasculitis mouse model. Current treatment options for KD include aspirin plus intravenous immunoglobulin (IVIG) therapy. However, the 20% of patients who do not respond to IVIG are at even increased risk of developing coronary artery aneurysms and cardiac sequelae^7^. IL-1R antagonist (Anakinra) is currently in clinical trials for IVIG-resistant KD patients, as human data also suggests that IL-1 plays an important role in KD^49^,^50^. Thus, quercetin may provide an alternative approach to prevent unwanted cardiovascular sequalae of KD that maybe due to over exuberant IL-1 signaling. Additionally, quercetin may also be a potential therapeutic candidate for CAPS and other inflammasome associated inflammatory diseases.

## Materials and Methods

### Mice

C57BL/6 mice were obtained from Jackson Laboratories (Bar Harbor, ME USA). *Nlrp3*^−/−^ mice were kindly provided by Dr. Fitzgerald (Univ. Massachusetts Medical School, MA USA). *Nlrp3*^A350V/350V^ (*Nlrp3*^A350V/+^ CreT) mice were kindly provided by Dr. Hoffman (UC San Diego, CA USA). *Atg16l1*^fl/fl^ mice were kindly provided by Dr. Shih (Cedars-Sinai Medical Center, CA USA) and bred with LysM^Cre^ mice (Jackson Laboratories). All mice used were males 8-12 weeks of age. All animals were housed under specific pathogen-free conditions at the animal center of the Cedars-Sinai Medical Center. Experiments were conducted under approved Institutional Animal Care and Use Committee protocols.

### Preparation of Lactobacillus cell wall extract and Kawasaki Disease mouse model

*L. casei* (ATCC 11578) cell wall extract (LCWE) was prepared as we previously described^51^. Four to five weeks aged male mice were injected intraperitoneally with 250 μg LCWE or PBS. Quercetin 100 mg/kg (treatment group) or 2% DMSO vehicle (control group) was administered daily i.p., 24 hours after LCWE injection. Mice were euthanized and hearts were removed at day 7 and embedded in optimal cutting temperature compound for histological examination as previously described^1^. Frozen abdominal aorta sections were immunohistochemically analyzed for macrophage, and caspase-1 activity using anti-mouse F4/80 (eBioscience, CA USA) and FLICA Caspase-1 (ImmunoChemistry Technologies LLC, MN USA), then mounted with DAPI (4,6-diamidino-2 phenylindole; Life Technologies, USA). IgG2a was used as the isotype control (AbD Serotec, OX UK). All images were obtained using a Keyence BZ-9000 fluorescent microscope (Keyence Corporation of America, IL USA).

### Cytokine Measurement

Bone marrow derived macrophages (BMDM) were prepared as previously described^52^. BMDM were stimulated with 500 ng/ml *E. coli* LPS (Invivogen, CA USA) and 3 h later treated with either 5 mM ATP (Sigma, MO USA), 10 μM nigericin (Enzo Life Science, INC., NY USA), or 130 μg/mL alum (Sigma) stimulation for NLRP3 inflammasome, 400 ng/ml poly(dA:dT) (Invivogen) for AIM2 inflammasome, and *Salmonella Typhimurium* IR715 *and* Δ*fljB/fliC* (MOI 5) for NLRC4 inflammasome activation^34^. BMDM were treated with quercetin, naringenin, or silymarin at the doses of 20 and 100 μM, 30 min before the signal-2 (ATP, nigericin, e.t.c.) stimulation. *Nlrp3*^A350/A350^ BMDM were primed with LPS (500 ng/mL) and treated with quercetin 1 hour after priming. Supernatants were collected and assessed for IL-1β and TNF-α concentration by ELISA (eBiosciences).

Quercetin, naringenin, and silymarin were purchased from Sigma. For *in vitro* experiments, a stock solution of the flavonoids dissolved in 100% dimethyl sulphoxide (DMSO) at the concentration of 200 mg/ml was further diluted with culture medium prior to administration in cell cultures. For *in vivo* treatment, quercetin (100 mg/kg) was first dissolved in 100% DMSO and then diluted with PBS to a final volume of 100 μl/10 g of body weight.

### Immunoblot assay

BMDM were stimulated as described above, supernatants were collected, and proteins were precipitated by methanol-chloroform extraction. Cell pellets were suspended in lysis buffer. Immunoblot analysis was performed using following primary antibodies: anti-mouse IL-1β (AF-401-NA; R&D System, MN USA), rabbit polyclonal anti-Caspase-1 (Santa Cruz Biotechnology), anti-β-actin (C4; Santa Cruz Biotechnology). For ASC oligomerization assay in lysates, cells suspension was washed with PBS and incubated with 2 mM disuccinimidyl suberate (DSS, No-Weigh™ Format, Pierce Protein Biology) for 30 minutes in room temperature. After washing with ice cold PBS, precipitates were suspended in lysis buffer. Immunoblot analysis was performed using rabbit anti-mouse ASC (N15; Santa Cruz Biotechnology). The supernatants were also used for anti-mouse ASC immunoblot and anti-phosphotyrosine (4G10; EMD Millipore, MA USA) immunoblot analysis. For immunoprecipitation, anti-phosphotyrosine agarose beads (PY20; EMD Millipore) were added into the supernatants and incubated for overnight, washed with lysis buffer for 3 times, and used for immunoblot with HRP conjugated rabbit anti-mouse ASC (LifeSpan Biosciences, Inc., WA USA). Image densitometry was performed using Image-Pro (MEDIA Cybernetics, INC., MD USA).

### Immunofluorescence staining

BMDM were plated at the density of 1 × 10^5^ cells/well in 24-well plate with a 0.2% gelatin pre-coated cover slip and stimulated as described above. One hour after stimulation cells were washed with ice-cold PBS, fixed in 1% formalin and permeabilized with 0.1% Triton X-100. After blocking with serum free Protein Block (Dako), cells were stained with primary rabbit anti-ASC (Santa Cruz Biotechnology), secondary Alexa Fluor 594 donkey anti-rabbit IgG (Invitrogen), then mounted with DAPI (Life Technologies). BDMD were separately stained with FLICA and mounted with DAPI. FLICA positive cells were analyzed. All images were obtained using a Keyence BZ-9000 fluorescent microscope (Keyence).

### Statistical analysis

All data were analyzed using Prism 5.0 statistical program (GraphPad software, Inc.). We used one-way ANOVA with Tukey’s *post hoc* test for analysis with three or greater groups. A *P* value less than 0.05 was considered statistically significant.

### Ethics statement

All animal experiments were performed according to the guidelines and approved protocol (IACUC Protocol #5093) of the Cedars-Sinai Medical Center Institutional Animal Care and Use Committee. Cedars-Sinai Medical Center is fully accredited by the Association for Assessment and Accreditation of Laboratory Animal Care (AAALAC International) and abides by all applicable laws governing the use of laboratory animals. Laboratory animals are maintained in accordance with the applicable portions of the Animal Welfare Act and the guidelines prescribed in the DHHS publication, Guide for the Care and Use of Laboratory Animals.

## Additional Information

**How to cite this article**: Domiciano, T. P. *et al*. Quercetin Inhibits Inflammasome Activation by Interfering with ASC Oligomerization and Prevents Interleukin-1 Mediated Mouse Vasculitis. *Sci. Rep.* **7**, 41539; doi: 10.1038/srep41539 (2017).

**Publisher's note:** Springer Nature remains neutral with regard to jurisdictional claims in published maps and institutional affiliations.
